# A Non-linear Predictive Model of Borderline Personality Disorder Based on Multilayer Perceptron

**DOI:** 10.3389/fpsyg.2018.00447

**Published:** 2018-04-04

**Authors:** Nelson M. Maldonato, Raffaele Sperandeo, Enrico Moretto, Silvia Dell'Orco

**Affiliations:** ^1^Department of Neuroscience, Reproductive and Odontostomatological Sciences, University of Naples Federico II, Naples, Italy; ^2^Scuola in Psicoterapia Gestaltica Integrata, Torre Annunziata, Italy

**Keywords:** borderline personality, neural network, character and temperament variables, dissociative phenomena, personality disorders

## Abstract

Borderline Personality Disorder is a serious mental disease, classified in Cluster B of DSM IV-TR personality disorders. People with this syndrome presents an anamnesis of traumatic experiences and shows dissociative symptoms. Since not all subjects who have been victims of trauma develop a Borderline Personality Disorder, the emergence of this serious disease seems to have the fragility of character as a predisposing condition. Infect, numerous studies show that subjects positive for diagnosis of Borderline Personality Disorder had scores extremely high or extremely low to some temperamental dimensions (harm Avoidance and reward dependence) and character dimensions (cooperativeness and self directedness). In a sample of 602 subjects, who have had consecutive access to an Outpatient Mental Health Service, it was evaluated the presence of Borderline Personality Disorder using the semi-structured interview for the DSM IV-TR personality disorders. In this population we assessed the presence of dissociative symptoms with the Dissociative Experiences Scale and the personality traits with the Temperament and Character Inventory developed by Cloninger. To assess the weight and the predictive value of these psychopathological dimensions in relation to the Borderline Personality Disorder diagnosis, a neural network statistical model called “multilayer perceptron,” was implemented. This model was developed with a dichotomous dependent variable, consisting in the presence or absence of the diagnosis of borderline personality disorder and with five covariates. The first one is the taxonomic subscale of dissociative experience scale, the others are temperamental and characterial traits: Novelty-Seeking, Harm-Avoidance, Self-Directedness and Cooperativeness. The statistical model, that results satisfactory, showed a significance capacity (89%) to predict the presence of borderline personality disorder. Furthermore, the dissociative symptoms seem to have a greater influence than the character traits in the borderline personality disorder e disease. In conclusion, the results seem to indicate that to borderline personality disorder development, contribute both psychic factors, such as temperament and character traits, and environmental factors, such as traumatic events capable of producing dissociative symptoms. These factors interact in a nonlinear way in producing maladaptive behaviors typical of this disorder.

## Introduction

Borderline Personality Disorder (BPD) is one of the major challenges for contemporary psychopathology as far as understanding its pathogenesis, clinical manifestations and treatment methods are considered. In terms of etiopathogenesis, substantial epidemiological (Ball and Links, 2009) evidence shows that subjects with BPD have traumatic events in their preceding anamnestic history. According to recent work (Meares, 2014), it would seem that BPD has many similarities with dissociative disorders regarding the difficulty that patients with both types of disorders have in regulating vegetative phenomena and emotional activation status. Other relevant research (Cloninger, 2000) from the point of view of the pathogenetic investigation revealed a relationship between BPD and extreme temperament and character trait expressions. For example, research (Pukrop, 2002; Joyce et al., 2010) conducted using the Cloninger Temperament and Character Test (TCI) showed that low levels of Self-Directedness and Cooperativeness and high levels of Harm Avoidance and Novelty Seeking are related to the presence of personality disorders described in Cluster B of the DSM-IV-TR (American Psychiatric Association, 1996).

Dissociative phenomena (Sperandeo et al., 2018), on the other hand, commonly conceptualized as an interruption in the usually integrated functions of consciousness (Maldonato, 2009, 2014; Maldonato et al., 2011), memory, identity and perception of the environment (DSM-IV), have been widely associated, in the same way as BPD (Korzekwa et al., 2009; Meares, 2014), traumatic events, Post-Traumatic Stress Disorder (PTSD) (Yager, 1976; Spiegel et al., 1988; Carlier et al., 1996), sexual abuse during childhood and deficit in parental care (Brodsky et al., 1995; Zlotnick et al., 1996).

It is useful to clarify that when it comes to “dissociation” there is not necessarily a reference to a clinically defined syndrome; in fact, this term in its current use, in scientific literature, refers to both clinically relevant symptomatic phenomena and adaptive mental processes (Spiegel et al., 2011; Cantone et al., 2012).

In addition, according to Grabe, dissociation is a phenomenon which is closely related to the personality dimension. Indeed, in a study based on the Cloninger's Seven Factor model (Svrakic et al., 1993), it was discovered that: in women and men, the temperament and character inventory dimensions of Self-Transcendence, Self-Directedness, were predictors for the Dissociative Experience Scale scores. No temperament dimensions showed any significant predictive power. Such evidence, according to the authors, would confine the genesis of dissociative phenomena to the context of events caused by environmental stresses (Grabe et al., 1999).

From the analysis of current literature, an expression ultimately emerges that expresses the close relationship between traumatic events, dissociative phenomena and the dimensions of temperament and character in the genesis of BPD. However, these relationships are clearly non-linear so that none of the phenomena described can be considered as the first or the essential etiologic mechanism. They seem rather tied to each other in a complex network characterized by overlapping areas and recursive mechanisms.

The currently acclaimed model of the Borderline Personality Disorder (BPD) pathogenesis shows that Traumatic environmental events, interacting with the structural fragility of a subject's personality trigger dysfunctional processes in the area of cognition, emotions, interpersonal relationships, and social behavior.

The interaction between the personality structure and the traumatic environmental events is a very articulate and complex process and, at the moment, only superficially touched by scientific enquiry. Studies have only succeeded in establishing correlations between traits of fragile personality, traumatic events and BPD; in some cases, it has also been possible to describe, through regression methods, the relationship between the phenomena discussed in this study, but the effect size of these relationships is always rather limited. Although these explanatory models are of great importance for the evolution of knowledge, their prediction capacity is still functionally inadequate.

Moreover, the question of the quality of the interaction between psychological and environmental processes is completely unrealistic at this stage; specifically, it is important to question how it proceeds and develops, what are the factors that enhance or inhibit it, and how and to what extent it is possible to predict the emergence of BPD following the collapse of individual resilience systems. This last question is extremely relevant also for the possibility of activating effective primary and secondary preventions interventions.

In this regard, it is useful to refer to the theory proposed by Brosboom as a methodology for exploring the non-linear interactive processes typical of psychological disorders. Network theory, a recent epistemological model in psychopathology (Borsboom and Cramer, 2013), allows to conceptualize mental disorders as the result of direct interactions between symptoms. The biological, psychological and social mechanisms to which the individual is exposed in his life experience, according to this model, activate the emergence of a specific symptom and this triggers a process of amplifying the network of related psychopathological phenomena. For example, an angiogenic environmental stimulus may induce an alteration in sleep-wake rhythm that, if persistent, triggers asthenia and demotivation, followed by other typical symptoms of depressive disease (Kendler, 2012). If the relationship between the symptoms is strong enough, they can generate a level of feedback such as self-feeding the system. Although this pattern of psychopathology is not universally accepted, it is an effective attempt to explain the non-linear relationships between the psychological phenomena underlying the pathogenetic mechanism of a disorder (Borsboom, 2017).

### Aims

The clinical relevance of BPD requires the search for effective prevention methods and hence the need for studies to gain not only certain evidence (as the current literature seems to outline) but also that it be materially usable in the development of instruments capable of predicting the onset of this disease with a clinically useful sensitivity and specificity.

The present study aims to investigate the quality of the pathogenetic process by improving the ability to predict, in a single subject, the BPD on the basis of the evaluation instruments applicable in ordinary clinical contexts. For this purpose, it is necessary to use statistical analysis instruments suitable for modeling non-linear phenomena that favor the ability to predict the emergence of BPD by the interaction between environment and psychological phenomena.

## Instruments

### Structured clinical interview for DSM (SCID-II)

SCID-II is an instrument used to diagnose personality disorders, both categorically (current or absent) or dimensionally. It consists of 119 items, having dichotomous answer modes Yes/ No. Every personality disorder is identified by a certain number of items that match DSM criteria for that specific diagnostic category. On the clinical interview model, the starting part consists of a brief overview that identifies the subject's normal behavior and relationships and allows to verify its introspection capabilities. A “3” score on a SCID-II item, provided by the clinician during the investigation, indicates that there is sufficient evidence that the feature described in the article is “pathological,” “persistent,” and “diffuse.” “Pathological” indicates that the characteristic is outside the range of normal variation; “Persistent” refers to both frequency and duration (a score of “3” means that the feature has been present frequently during the last 5 years); “Diffused” indicates the presence of the feature in various contexts, such as at home and at work, or, in the case of items relating to interpersonal relationships, it comes out in different relationships. The interview was conducted by 5 trained researchers who achieved a high inter-rater reliability (*k* = 0.81) (First et al., 1997; Maffei et al., 1997).

### Dissociative experience scale (DES)

DES is a self-report instrument for rapid compilation and processing that can assess the presence, quantity, and type of dissociative experiences without entering into the merit of diagnosis. It consists of 28 items arranged on an analog scale and scores vary from 0 to 100, for each item, and for the total score obtained from the average of the scores. The cut-off value indicating the presence of pathological dissociation concerns scores ≥20: scores greater than 20 are generally associated with a DSM-IV-TR dissociative disorder diagnosis, lower scores are commonly observed in both healthy subjects and in psychiatric patients in general.

The factorial analysis provided an understanding of the DES structure: for the Italian version, the three-factor model is the most frequently used one. According to this model, DES is composed of the following sub-scale:

- Dissociative Amnesia, which concerns actions of which the subject has no memory (item: 3, 4, 5, 8, 11);
- Imaginative Absorption And Involvement, indicating total immersion into an activity so as to become completely unaware of the surrounding environment (items 2, 14, 15, 17, 18, 20, 24);
- Depersonalization-Derealization, or altered perceptions of the self and the environment, such as feeling disconnected from one's own body, from their own thoughts, from their feelings (items 7, 12, 13, 21, 22, 23, 27, 28).

Subsequently, Waller et al. have selected 8 items of DES (item: 3, 5, 7, 8, 12, 13, 22, 27) by building a new subcategory that can identify the tendency to pathological dissociation, DEStax (Waller et al., 1996).

DES is a valid and reliable instrument for measuring dissociative experiences both in clinical and control samples and reveals a similar factorial structure in groups of psychiatric patients and normal subjects. Scores at DES-TOT over 20 are considered indicative of a pathological condition, but have no diagnostic value (Bernstein and Putnam, 1986; Fabbri Bombi et al., 1996).

### Temperament and character inventory (TCI)

In order to come up with a diagnosis according to the Cloninger model, the instrument that the same author has developed can be used: Temperament and Character Inventory (TCI). The TCI is a self-report (self-report) questionnaire that, in its most complete version, is composed of 240 items in response to a dichotomy (true/false). Of these, 116 explore the 4 temperamental dimensions (NS, HA, RD, and P), 119 evaluate the 3 character dimensions (SD, C, and ST) and 5 are indicators of the presence of Personality Disorders. The sum of the items marked as “true” provides the raw scores of the seven scales. The raw scores are transformed into T standardized scores that, shown on a graph, provide a personality profile of the subject. This instrument includes questions about tastes, interests, emotions, responses, goals, and values. TCI results can be evaluated as raw score, T score, and scoring percentage, and a conversion table is provided between these three measures based on the score obtained in a standardization on a sample of 300 adults called a community sample; Cloninger says that this is representative of the general population and supports the reliability and structure of the TCI size. Temperament is considered to be the emotional heart of the personality. It includes four largely independent dimensions: (1) Novelty Seeking (NS), which represents behavioral activation in response to novelty and reward or punishment relief; (2) Harm Avoidance (HA), referring to behavioral inhibition in response to punishment or non-reward signals; (3) Reward Dependence (RD), reflecting socially rewarded behaviors; (4) Persistance (P) which describes persevering behavior despite fatigue and frustration. Character, however, is defined in terms of individual differences in the concept of self-experience that evolves throughout life in response to socio-cultural influences. It includes three dimensions: (1) Self Directedness (SD), which is the ability to adjust and adapt behavior to the needs of a situation in order to achieve personally chosen goals; (2) Cooperativeness (C), which expresses self-identification as an integral part of a more or less inclusive social group and the level of liking in relationships with others; (4) Self Transcendence (ST), which is associated with the ability to recall the past and to imagine the future as the evolution of life's history, as well as experiencing unity with nature and developing spiritual values (Cloninger et al., 1993).

## Sample

The sample consists of 602 subjects of which 160 are diagnosed with Borderline Personality Disorder (BPD). The subjects belonging to the sample had an average age of 34.7 years of age with a standard deviation of 11.48; 266 were males and 336 were females; 42 subjects had completed elementary school, 119 had completed middle school, 326 had achieved high school diploma, 114 were graduates and only one participant lacked any qualification. Regarding the civil status, 327 persons are singles, 226 are married, 34 are separated or divorced, 15 are widowed. Finally, 419 subjects are employed in work or study, 174 are unemployed and 9 are retired.

## Methods

To achieve the aim of the study a feed-forward neural network type, that evolves through a process called Error Back Propagation (EBP), was used. This type of neural network called Perceptron Multilayer function as a powerful interpolation mathematical “trainer” system capable of calculating non-linear functions of any complexity from known input and output values used as examples for learning (Rumelhart et al., 1985).

**Figure 1:**
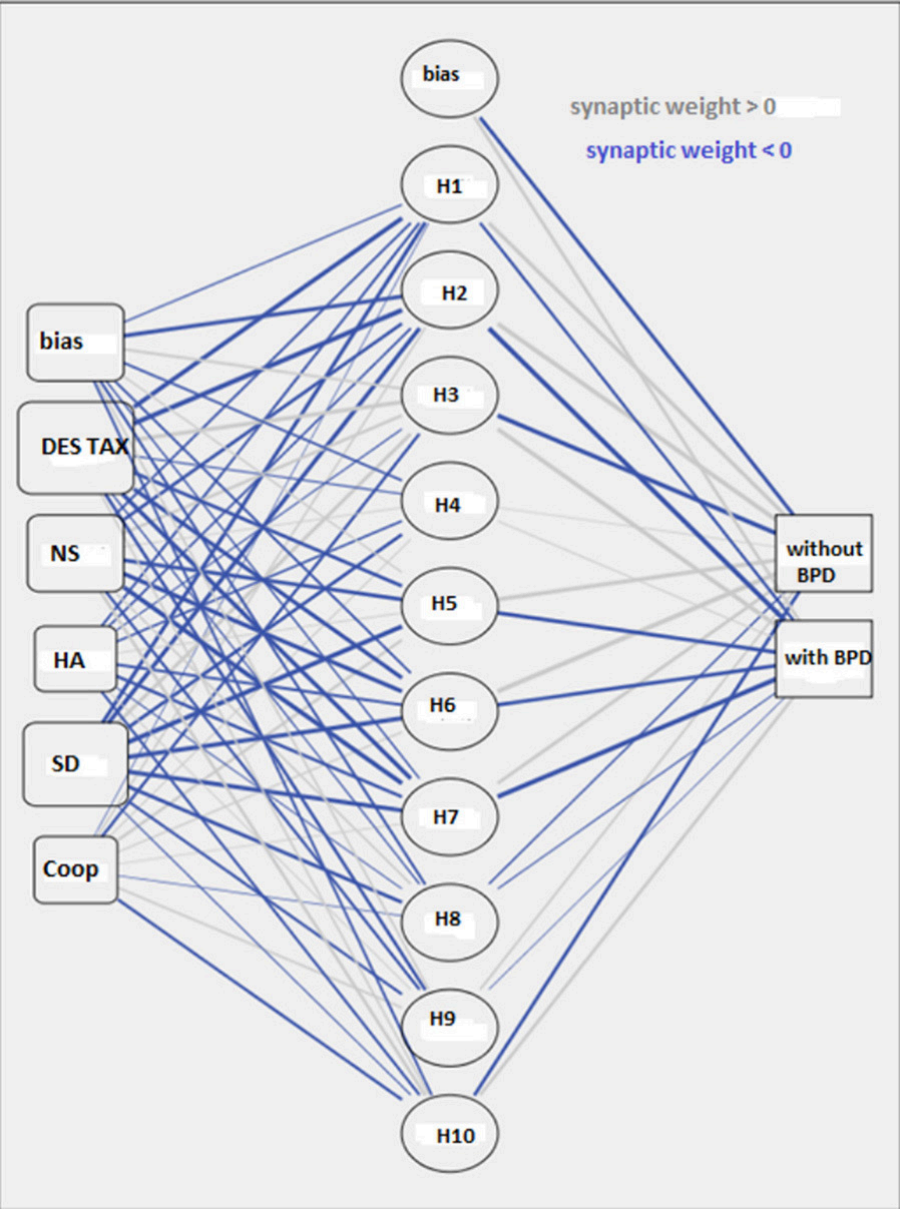
Graph of the multilayer perceptron network.

Figure 1 depicts the Perceptron Multilayer Network EBP used in the study. This consisting of: an input layer of 5 input variables, of a hidden layer consisting of 10 nodes of computation and an output layer of consisting of two output nodes.

Two types of assessment were used as input variables; on the one hand, measurements (obtained with TCI) of the intensity of expression of the temperament and character traits that in the pertaining literature were systematically correlated with BPD, on the other the measurement (obtained with the DES) of the intensity of the dissociative experiences. When these measures present values below or above certain thresholds, they describe the presence of character and temperament fragility and of abnormal reactions to traumatic events. Specifically, scores above 70° percentiles as per the TCI temperamental scales, described as “Novelty Seeking” (NS) and “Harm Avoidance” and scores below 30° percentiles as per the character scales described as “Self Directiveness” (SD) and “Cooperativeness” (Coop) have been correlated to the presence of BPD in the same way as the high scores have been correlated to the taxonomic factor of the DES (DEStax) (Meares, 2014).

The scores obtained in these specific scales, from subjects belonging to the sample being examined, were transformed in continuous values comprised between −1 and 1 with a procedure known as “feature scaling.” Adjusted version of subtracting the minimum and dividing by the range (2^*^(x –min)/(max–min)) −1. Adjusted normalized values fall between −1 and 1. These values represent the input signals conducted by the nodes of the first layer, which, for this purpose, were labeled with the names of the 5 scales described above NS, HA, SD, Coop e DEStax).

Each of the 10 hidden layer computing units labeled with the letter H and an order number between 1 and 10 received, as an input, the scores of each of the 5 variables of the first layer. Each H node has produced two output values one for each of the two nodes of the third layer (Y), these nodes process information regarding the presence of BPD and are therefore labeled “with BPD” and “without BPD.”

Each value of the neurons H and of the neurons Y has been associated with a weight “w” which is a multiplicative factor applied to the input signal; each node is associated with a bias “wb.” The weighed sum of all the input signals of the nodes (H and Y) defines the internal activation of node named “A.”

For example, in the case of the third output layer node “with BPD” the internal activation “A” is described by the following formula

Ay, withBPD = H1w1 + H2w2.…+ H10w10 + wby, withBPD

In the case of the node of the second layer “H1” the internal activation “A” is described by the following formula

AH1 = DEStaxwdestax + NSwns + HAwha + SDwsd + Coopwcoop + wbH1

The output signal of the nodes H and Y, defined as Neuronal Activity “AN,” was calculated by applying a sigmoid-shaped transfer function

AN = 1/1 + e-A

Where “e” is the basis of the natural logarithms (2.71828) and A and the internal activity of the node.

**Figure 2:**
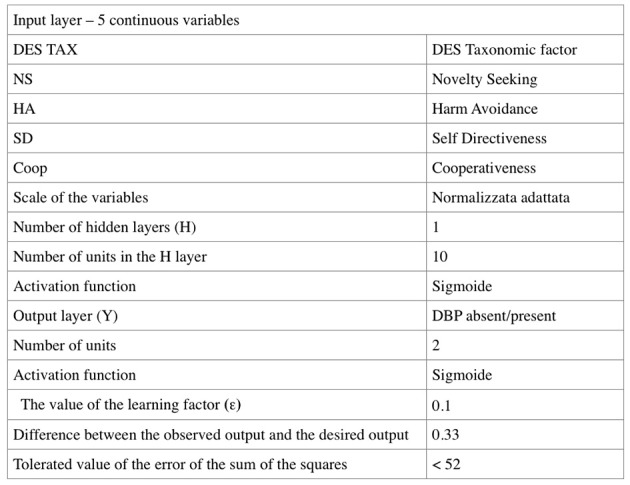
Design of the multilayer perceptron network.

For network training (Figure 2), the synapse weights were set to small random values; a training sample of 80% of the total sample was defined; the scores obtained from this subgroup of subjects to the 5 variables of TCI and DES were used to provide input data to the network; as output values coupled to input variables, the presence (with BPD) or absence (without BPD) of Borderline Personality Disorder Diagnosis was used. The core of the learning algorithm EBP is represented by a method of minimizing the error defined as “steepest descendent method” (Courant, 1943).

In the Back-Propagation phase, a “Batch” type weight correction process was applied in which all corrections are made at the end of all subgroup training cycles by using all the information of the training subjects. A repetition of the correction process was planned so as to obtain an effective output that did not deviate by more than 0.33 for each sample from the sample output used for training.

Each weight has been altered by an amount equal to the delta of the neuron multiplied by the input value on that connection, which is multiplied by an Epsilon (ϵ) factor, labeled learning factor equal to 0.1 selected to obtain a high number of epochs of learning which would guarantee a more accurate model and avoid the risk of a local minimum disabling the process.

Training of the Intermediate neurons was done by back-propagation through the delta error that had been calculated on the nodes of the third layer.

Finally, because the connection proceeds to several neurons of the next layer the error was calculated as the sum of all the individual Δ weighed. Calculation of the learning and verification Phase, model description, discrimination analysis, ROC Curves and analysis of the importance of the variables were performed with the help of the Statistical Package for Social Science (SPSS) software. This computer instrument, even assuming the initial weight values in random order, is able to repeat the data by setting the random number generator to an initial fixed value.

## Analysis of the results

**Figure 3:**
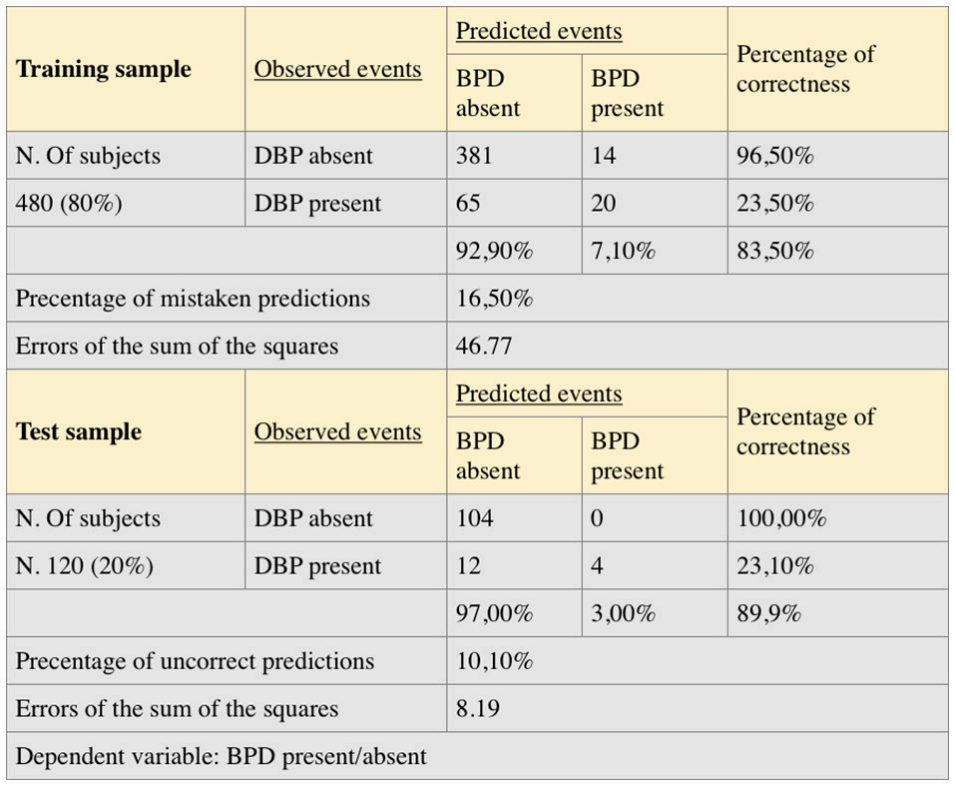
Summary of the model and discriminant analysis.

Figure 3 reviews the summary of the regression model produced by the multilayer perceptron network where the percentage of correctness of the prediction of the presence and absence of the BPD are assessed; the model appears valid in terms of statistical reliability.

The learning process developed on a group of 480 subjects which represent 80% of the total sample (described at the top of the table) stopped after less than 100 cycles because it reached an error level below the selected value of 0.33. This value was chosen because the approximation of less than 33% to the output value 1 that expresses BPD is sufficient to guarantee the accuracy of the forecast. This value of tolerated difference between the desired output and the output observed in the single case allows us to calculate the accepted value of the error of sum of squares according to the following formula:

$$\begin{array}{l} \text{E }=\;\left(1/2\right)\text{KNe} \end{array}$$

where E = error of the sum of squares, K = number of cases used for learning, N = number of output nodes, e = tolerated difference between the desired value and the value observed in the individual case (Pessa, 2004).

$$\begin{array}{l} \left(1/2\right)\cdot 480\cdot 2\cdot 0.332\;=\;52 \end{array}$$

The error of the sum of squares has reached a value of 46.77 at the end of the training phase. The percentage of incorrect predictions in the learning sample was 16.5%. The test sample (described in the lower part of Figure 3) is made up of 120 subjects. In this sample, the sum of squares has reached a value of 8.19 and the percentage of incorrect predictions was 10.1%.

**Figure 4:**
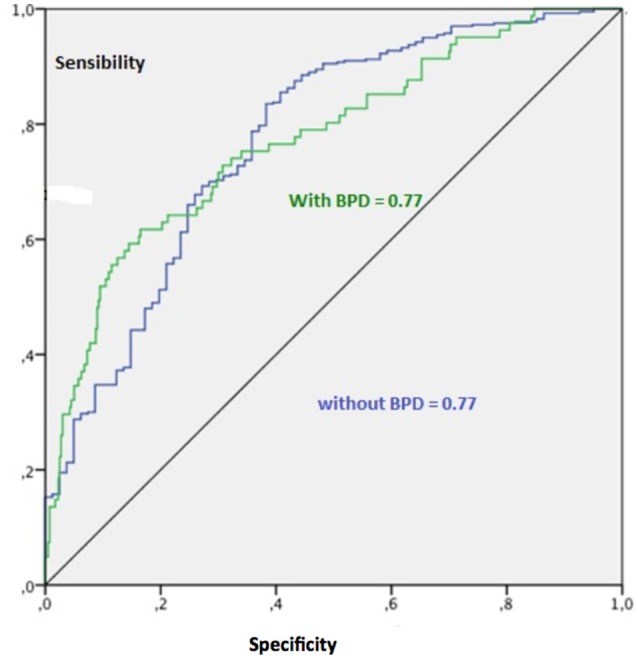
Curves receiver operating characteristic (ROC).

**Figure 5:**
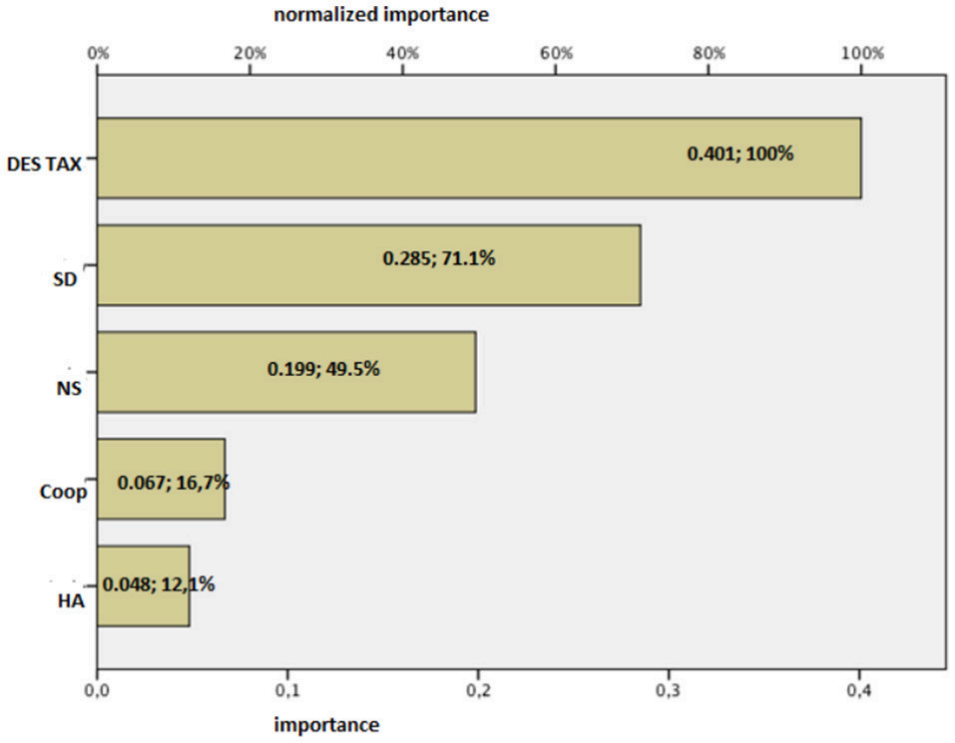
Importance of the independent variables.

Figure 4 shows the ROC curves for the sensitivity and specificity of the model in predicting the presence and an absence of diagnosis of BPD. The value of the area under the curve of 0.77 expresses a good sensitivity of the model. Figure 5 shows the analysis of the importance of the variables (Towell and Shavlik, 1992), which shows that high scores at the taxonomic scale of DES and Novelty Seeking scale have a weight of 40 and 20% respectively in the model in question, while Self-Directiveness scales have a weight of 28.5% in determining the model's prediction accuracy.

## Discussion

The study data describes a non-linear relationship between the variables in question and show how in the genesis of BPD, the presence of dissociative phenomena has a double weight of character and temperament variables (SD and NS). DES is an instrument that detects dissociative phenomena rather than their pathological nature. The taxonomic scale (DEStax) correlates more than the other scales with dissociative syndromes classified in DSM. However, it does not consent to formulate any diagnosis.

Subjects with high scores at DEStax are not necessarily affected by dissociative disorders and often exhibit a mode that is biologically determined to respond to traumatic events that that they seem to manipulate by means of detachment, amnesia or imaginative absorption. From our study, it can be seen that when these subjects, who have an anomalous reactivity to trauma, have a marked explorative tendency or poor ability in self-determination functions because of their life experiences, there is a 90% probability that they can develop a BPD.

As proposed by Borsboom, the pathology develops on the basis of an interaction between psychopathological processes; he specifies that this interaction concerns the individual symptomatic level so the nodes of the network are internal to the pathology itself (Jones et al., 2017).

Our study said that, at least as regards personality disorders (Sperandeo et al., 2016), nonlinear interaction is also about pre-pathological phenomena such as extreme expressions of dissociative dimensions and personality, as many psychic phenomena as possible and environmental events. This integrative vision also emerges also in the commentary by Jones et al., on the work of Borsboom. These authors propose “an expansion of the network theory of psychopathology in which nodes consist of individual level causal variables.” Expanding the network approach beyond symptoms will further strengthen this potentially revolutionary framework for studying psychopathology. These evidences confirm the validity of the intuitions of Borsboom with about the utility of imagining a psychopathological theory of networks. However, in our opinion it is important to integrate descriptive elements of psychopathological functioning and environmental stressors into this theoretical vision.

The dimensions of Temperament and Character detected by the TCI and the dissociative experiences described by DES are phenomenological, methodologically and theoretically different descriptors from the SCID items. In fact, they do not describe symptoms but ordinary processes that, on the one hand do not activate defense mechanisms in users (who tend to reject symptomatic stigmatization) and on the other hand are detectable before the symptoms actually arise.

The usefulness of a model that approaches the development of BPD starting from pre-symptomatic psychic processes is evident in the possibility of creating predictive tools for the future development of the disease. Knowledge of the form of the interaction between personality dimensions and life experiences is essential to implement secondary prevention interventions.

It is necessary to apply the model to larger test samples to confirm the predictive validity of the same; subsequently it will be possible to integrate it with descriptive dimensions of the environmental experience and of the more refined and specific character processes in order to derive the description of the etiopathogenetic interaction from the model. In the future perspective we intend to integrate the predictors of the pathology obtaining a complete pattern psychical and environmental conditions significant for the pathology is to activate follow-up studies to test the model's ability to predict the development of disorders.

## Ethics statement

This study was carried out in accordance with the recommendations of codice etico per la ricerca in psicologia, comitato etico Associazione Italiana di Psicologia with written informed consent from all subjects. All subjects gave written informed consent in accordance with the Declaration of Helsinki. The protocol was approved by the comitato etico per la ricerca della SiPGI Postgraduate School in Gestalt Integrated Psychotherapy D.M.I.U.R. 12.10.2007.

## Author contributions

All authors listed, have made substantial, direct and intellectual contribution to the work, and approved it for publication.

### Conflict of interest statement

The authors declare that the research was conducted in the absence of any commercial or financial relationships that could be construed as a potential conflict of interest. The reviewer, EP and handling Editor declared their shared affiliation.

## References

[B1] Ball J. S. Links P. S. (2009). Borderline personality disorder and childhood trauma: evidence for a causal relationship. Curr. Psychiatry Rep. 11, 63–68. 10.1007/s11920-009-0010-4 19187711

[B2] Bernstein E. M. Putnam F. W. (1986). Development, reliability, and validity of a dissociation scale. J. Nerv. Mental Dis. 174, 727–735. 3783140 10.1097/00005053-198612000-00004

[B3] Borsboom D. (2017). A network theory of mental disorders. World Psychiatry 16, 5–13. 10.1002/wps.20375 28127906 PMC5269502

[B4] Borsboom D. Cramer A. O. (2013). Network analysis: an integrative approach to the structure of psychopathology. Annu. Rev. Clin. Psychol. 9, 91–121. 10.1146/annurev-clinpsy-050212-185608 23537483

[B5] Brodsky B. S. Cloitre M. Dulit R. A. (1995). Relationship of dissociation to self-mutilation and childhood abuse in borderline personality disorder. Am. J. Psychiatry 152, 1788–1792. 10.1176/ajp.152.12.1788 8526247

[B6] Cantone D. Sperandeo R. Maldonato M. N. Cozzolino P. Perris F. (2012). Fenomeni dissociativi in un campione di pazienti ambulatoriali. Riv. Psichiatr. 47, 246–253. 10.1708/1128.12448 22825441

[B7] Carlier I. V. Lamberts R. D. Fouwels A. J. Gersons B. P. (1996). PTSD in relation to dissociation in traumatized police officers. Am. J. Psychiatry 153, 1325–1328 10.1176/ajp.153.10.1325 8831442

[B8] Cloninger C. R. (2000). A practical way to diagnosis personality disorder: a proposal. J. Pers. Disord. 14, 99–108. 10.1521/pedi.2000.14.2.99 10897461

[B9] Cloninger C. R. Svrakic D. M. Przybeck T. R. (1993). A psychobiological model of temperament and character. Arch. Gen. Psychiatry 50, 975–990. 8250684 10.1001/archpsyc.1993.01820240059008

[B10] Courant R. (1943). Variational methods for the solution of problems of equilibrium and vibrations. Bull. Am. Math. Soc. 49, 1–23. 10.1090/S0002-9904-1943-07818-4

[B11] Fabbri Bombi A. Bertin I. Cristante F. Colombo F. (1996). Un contributo alla standardizzazione della Dissociative Experiences Scale (DES) di Bernstein e Putnam. Bollettino di Psicologia Applicata 219, 39–46.

[B12] First M. B. Gibbon M. Spitzer R. L. Benjamin L. S. (1997). User's Guide for the Structured Clinical Interview for DSM-IV Axis II Personality Disorders: SCID-II. Washington, DC: American Psychiatric Pub.

[B13] Grabe H. J. Spitzer C. Juergen Freyberger H. (1999). Relationship of dissociation to temperament and character in men and women. Am. J. Psychiatry 156, 1811–1813. 10.1176/ajp.156.11.1811 10553748

[B14] Jones P. J. Heeren A. McNally R. J. (2017). Commentary: a network theory of mental disorders. Front. Psychol. 8:1305. 10.3389/fpsyg.2017.01305 28824490 PMC5539126

[B15] Joyce P. R. Light K. J. Rowe S. L. Cloninger C. R. Kennedy M. A. (2010). Self-mutilation and suicide attempts: relationships to bipolar disorder, borderline personality disorder, temperament and character. Aust. N. Z. J. Psychiatry 44, 250–257. 10.3109/00048670903487159 20180727

[B16] Kendler K. S. (2012). The dappled nature of causes of psychiatric illness: Replacing the organic–functional/hardware–software dichotomy with empirically based pluralism. Mol. Psychiatry 17, 377–388. 10.1038/mp.2011.182 22230881 PMC3312951

[B17] Korzekwa M. I. Dell P. F. Pain C. (2009). Dissociation and borderline personality disorder: an update for clinicians. Curr. Psychiatry Rep. 11, 82–88. 10.1007/s11920-009-0013-1 19187714

[B18] Maffei C. Fossati A. Agostoni I. Barraco A. Bagnato M. Deborah D.. (1997). Interrater reliability and internal consistency of the structured clinical interview for DSM-IV axis II personality disorders (SCID-II), version 2.0. J. Pers. Disord. 11, 279–284. 10.1521/pedi.1997.11.3.279 9348491

[B19] Maldonato N. M. (2009). From neuron to consciousness: for an experience-based neuroscience. World Futures 65, 80–93. 10.1080/02604020802594857

[B20] Maldonato M. (2014). The ascending reticular activating system, in Recent Advances of Neural Network Models and Applications. Smart Innovation, Systems and Technologies, Vol. 26, eds Bassis S. Esposito A. Morabito F. (Cham: Springer), 333–344.

[B21] Maldonato N. M. Dell'Orco S. Springer M. (2011). Rethinking consciousness: some hypothesis on the role of the ascending reticular activating system in the global workspace, in WIRN (Amsterdam), 212–219.

[B22] Meares R. (2014). Un Modello Dissociativo del Disturbo Borderline di Personalità. Milano: Raffaello Cortina Editore.

[B23] Pessa E. (2004). Statistica con le Reti Neurali: un'Introduzione. Roma: Di Renzo.

[B24] American Psychiatric Association (1996). DSM-IV Manuale Diagnostico e Statistico dei Disturbi Mentali. Milano: Ed. Masson.

[B25] Pukrop R. (2002). Dimensional personality profiles of borderline personality disorder in comparison with other personality disorders and healthy controls. J. Pers. Disord. 16, 135–147. 10.1521/pedi.16.2.135.22550 12004490

[B26] Rumelhart D. E. Hinton G. E. Williams R. J. (1985). Learning Internal Representations by Error Propagation (No. ICS-8506). San Diego, CA: California University San Diego, La Jolla Institute for Cognitive Science 10.21236/ADA164453

[B27] Sperandeo R. Esposito A. Maldonato M. Dell'Orco S. (2016). Analyzing correlations between personality disorders and frontal functions: a pilot study, in Advances in Neural Networks. Smart Innovation, Systems and Technologies, Vol. 54, eds Bassis S. Esposito A. Morabito F. Pasero E. (Cham: Springer), 293–302.

[B28] Sperandeo R. Monda V. Messina G. Carotenuto M. Maldonato N. M. Moretto E.. (2018). Brain functional integration: an epidemiologic study on stress-producing dissociative phenomena. Neuropsychiatr. Dis. Treat. 14, 11–19. 10.2147/NDT.S146250 29296086 PMC5741075

[B29] Spiegel D. Hunt T. Dondershine H. E. (1988). Dissociation and hypnotizability in posttraumatic stress disorder. Am. J. Psychiatry 145, 301–305 10.1176/ajp.145.3.301 3344845

[B30] Spiegel D. Loewenstein R. J. Lewis-Fernández R. Sar V. Simeon D. Vermetten E. (2011). Dissociative disorders in DSM-5. Depress. Anxiety 28, E17–E45. 10.1002/da.20923 22134959

[B31] Svrakic D. M. Whitehead C. Przybeck T. R. Cloninger C. R. (1993). Differential diagnosis of personality disorders by the seven-factor model of temperament and character. Arch. Gen. Psychiatry 50, 991–999. 10.1001/archpsyc.1993.01820240075009 8250685

[B32] Towell G. Shavlik J. W. (1992). Interpretation of artificial neural networks: mapping knowledge-based neural networks into rules, in Advances in Neural Information Processing Systems (San Mateo, CA), 977–984.

[B33] Waller N. Putnam F. W. Carlson E. B. (1996). Types of dissociation and dissociative types: a taxometric analysis of dissociative experiences. Psychol. Methods 1, 300–321. 10.1037/1082-989X.1.3.300

[B34] Yager J. (1976). Psychiatrically maladjusting soldiers. Arch. Gen. Psychiatry 33, 1332–1335. 10.1001/archpsyc.1976.01770110060005 985044

[B35] Zlotnick C. Shea M. T. Pearlstein T. Simpson E. Costello E. Begin A. (1996). The relationship between dissociative symptoms, alexithymia, impulsivity, sexual abuse, and self-mutilation. Compr. Psychiatry 37, 12–16. 10.1016/S0010-440X(96)90044-9 8770520

